# Bayesian Estimation of Combined Accuracy for Tests with Verification Bias

**DOI:** 10.3390/diagnostics1010053

**Published:** 2011-12-15

**Authors:** Lyle D. Broemeling

**Affiliations:** Broemeling & Associates Inc., 1023 Fox Ridge Road, Medical Lake, WA 99022, USA; E-Mail: broemeli2@aol.com

**Keywords:** Bayesian, inverse probability weighting, verification bias, risk score, combined test accuracy

## Abstract

This presentation will emphasize the estimation of the combined accuracy of two or more tests when verification bias is present. Verification bias occurs when some of the subjects are not subject to the gold standard. The approach is Bayesian where the estimation of test accuracy is based on the posterior distribution of the relevant parameter. Accuracy of two combined binary tests is estimated employing either “believe the positive” or “believe the negative” rule, then the true and false positive fractions for each rule are computed for two tests. In order to perform the analysis, the missing at random assumption is imposed, and an interesting example is provided by estimating the combined accuracy of CT and MRI to diagnose lung cancer. The Bayesian approach is extended to two ordinal tests when verification bias is present, and the accuracy of the combined tests is based on the ROC area of the risk function. An example involving mammography with two readers with extreme verification bias illustrates the estimation of the combined test accuracy for ordinal tests.

## 1. Introduction

This article introduces the reader to the methodology of measuring the accuracy of several medical tests that are administered to the patient. Our main focus is on measuring the accuracy of a combination of two or more tests. For example, to diagnose type 2 diabetes, the patient is given a fasting blood glucose test, which is followed by an oral glucose tolerance test. What is the accuracy (true and false positive fractions) of this combination of two tests? Or, in order to diagnose coronary artery disease, the subject's history of chest pain is followed by an exercise stress test. Still another example is for the diagnosis of prostate cancer, where a digital rectal exam is followed by measuring PSA (prostate specific antigen). The reader is referred to Johnson, Sandmire, and Klein [1] for a description of additional examples of multiple tests to diagnose a large number of diseases, including heart disease, diabetes, lung cancer, breast cancer, *etc.*

In many scenarios, it is common practice to administer one or more tests to diagnose a given condition and we will explore two avenues. One case where it is common to administer several tests is in standard medical practice, and the other is an experimental situation where one test is compared to a standard medical test. An example of the latter is that MRI is now being studied as an alternative to standard mammography, as a means to diagnose breast cancer.

There are many studies that assess the accuracy of the combination of two or more tests. Two tests for the diagnosis of a disease measure different aspects or characteristics of the same disease. In the case of diagnostic imaging, two modalities have different qualities (resolution, contrast, and noise), thus, although they are imaging the same scene, the information is not the same from the two sources. When this is the case, the accuracy of the combination of two modalities is of paramount importance. For example, the accuracy of the combination of mammography and scintimammography for suspected breast cancer has been reported by Buscombe, Cwikla, Holloway, and Hilson [2]. Another study for diagnosing breast cancer was performed by Berg, Gutierrez, *et al.* [3] who measured the accuracy of mammography, clinical examination, ultrasound, and MRI in a preoperative assessment of the disease, The accuracy of each modality and various combinations of the modalites were measured. When investigating metastasis to the lymph nodes in lung cancer, Van Iverhagen, Brakel, and Heijenbrok *et al.* [4] measured the accuracy of ultrasound and CT and the combination of two. Ultrasound conveys different information about metastasis compared to CT, but the combination of the two might provide a more accurate diagnosis than each separately. For an example of the diagnosis of head and neck cancer, Pauleit, Zimmerman, Stoffels *et al.* [5] used two nuclear medicine modalities, ^18^F-FET PET and ^18^F-FDG PET to assess the extent of the disease and estimated the accuracy of each and combined. On the other hand, Schaffler, Wolf, Schoelinast *et al.* [6] evaluated pleural abnormalities with CT and ^18^F-FDG PET and the combination of the two.

## 2. Measuring the Combined Accuracy of Two Binary Tests

What is the optimal way to measure the accuracy for combination of two binary tests? Pepe ([7], p. 207) presents two approaches: (1) believe the positive rule, or BP, where a positive test score on a subject occurs when one or the other of the two tests is scored positive; and (2) believe the negative rule, or BN, where a subject is scores positive if both tests are scored positive. Pepe also provides some properties about these rules, namely:

- The BP rule increases sensitivity relative to the two binary tests, but increases the FPF (false positive fraction), but by no more than the sum of the two false positive fractions FPF1 + FPF2. The false positive fraction of test 1 and test 2 are denoted by FPF1 and FPF2 respectively;
- The BN rule decreases the false positive rate relative to the false positive rates of the two tests, but at the same time, decreases the sensitivity, however, the sensitivity remains above TPF1 + TPF2 − 1. Note that the true positive fractions for the two tests are designated by TPF1 and TPF2.

Thus, with the BP rule the combined test is scored positive if one or the other or both of the two are scored positive, but, on the other hand, with the BN rule the combined test is scored positive if both tests are positive.

## 3. Verification Bias

Verification bias is present when some of the subjects are not subject to the gold standard, thus, the disease status of some of the patients is not known. Consider an example involving the exercise stress test to diagnose heart disease. Among those that test positive, some will undergo coronary angiography to confirm the diagnosis. On the other hand, among those that test negative, very few will be subject to the gold standard. Using only those cases that are verified will lead to biased estimates of test accuracy both of the true and false positive rates, thus, alternative methods based on the missing at random assumptions will be derived from a Bayesian viewpoint.

When assessing the accuracy of two tests, the design in many cases is paired. When two tests are used to assess the patient's condition, true and false positive fractions will be estimated assuming the BP (believe the positive) and BN (believe the negative) rules. For example, two modalities (e.g., CT and MRI) are imaging the same patients and the two images would be expected to be quite similar. Another case of a paired design is for two readers who are imaging the same set of patients with the same imaging device. One expects the information gained from the two paired sources to be highly correlated, and in the case of two paired readers, agreement between the two is also of interest. The experimental layout for a paired study when verification bias is present, namely:

A Good introduction to statistical methods for estimating test accuracy with verification bias is Zhou *et al.* ([8], p. 307), who use maximum likelihood estimation for the study below.

The two binary tests are *Y*_1_ and *Y*_2_, where 1 and 0 designate positive and negative tests respectively. For those subjects who are verified for disease V = 1, while those who are not verified are denoted with V = 0.

Note that the number of subjects verified under the gold standard, when both tests are positive, is *s*_11_ + *r*_11_, among which *s*_11_ had the disease and *r*_11_ did not have the disease, and also the number that were not verified under the gold standard when both tests are positive is *u*_11_ *etc.*, also note that the total number of subjects is $\overset{i,j=1}{\underset{i,j=0}{\text{∑}}}m_{ij}=m_{‥}.$

## 4. Posterior Distribution of Combined Test Accuracy

The following derivation is based on the MAR assumption, namely

$$P[V=1|Y_{1},Y_{2},D]=P[V=1|Y_{1},Y_{2}]$$

Thus, the probability a subject's disease status is verified depends only on the outcomes of the two tests, and not on other considerations. Derivations below follow to some extent Chapter 10 of Zhou, Obuchowski, and McClish^8^.

Suppose the unknown parameters are defined as follows:

(1) $$\varphi_{ij}=P[D=1|Y_{1}=i,Y_{2}=j]$$

and

(2) $$\theta_{ij}=P[Y_{1}=i,Y_{2}=j]$$

for i, j = 0, 1.

Also let

(3) $$\varphi_{i.}=P[D=1|Y_{1}=i]$$

and

(4) $$\varphi_{.j}=P[D=1|Y_{2}=j]$$

where i, j = 0, 1.

The likelihood for the parameters is

(5) $$L(\theta ,\varphi )\propto \prod_{i=0}^{i=1}\prod_{j=0}^{j=1}\varphi_{ij}^{s_{ij}}{\left(1-\varphi_{ij}\right)}^{r_{ij}}\prod_{i=0}^{i=1}\prod_{j=0}^{j=1}\theta_{ij}^{m_{ij}}$$

Assuming an improper prior distribution for the parameters, the posterior distributions are

(6) $$\varphi_{ij}\sim \text{beta}(s_{ij},r_{ij})$$

for i, j = 0, 1, and the *θ_ij_* are distributed Dirichlet with parameter vector (*m*_00_, *m*_01_, *m*_10_, *m*_11_).

The improper prior imposed on the parameters is given by the density

$$\xi (\theta ,\varphi )\propto \prod_{i=0}^{i=1}\prod_{j=0}^{j=1}\theta_{ij}^{-1}\varphi_{ij}^{-1}$$

Note that

$$\varphi_{1.}\sim \text{beta}(s_{1.},r_{1.})$$

and

(7) $$\varphi .1\sim \text{beta}(s_{.1},r_{.1})$$

where

$$s_{1.}=s_{11}+s_{10}$$

and

$$r_{1.}=r_{11}+r_{10}$$

Note that if a uniform prior is assumed, one should adjust the posterior distributions for the phis and thetas by adding a one to the beta and Dirichlet hyper parameters given in formulas (6) and (7).

The main parameters of interest are the true positive fraction and the false positive fraction for the two tests, thus for the first test

$$tpf_{1}=P[Y_{1}=1|D=1]$$

and is given by Bayes theorem as

(8) $$tpf_{1}=\varphi_{1.}\theta_{1.}/(\varphi_{1.}\theta_{1.}+\varphi_{0.}\theta_{0.})$$

where the *ϕ_i._* are given by (8.19) and

$$\theta_{1.}=\theta_{11}+\theta_{10}$$

As for test 1, the false positive fraction is given by

(9) $$fpf_{1}=(1-\varphi_{1.})\theta_{1.}/(1-\varphi_{1.}\theta_{1.}-\varphi_{0.}\theta_{0.})$$

With regard to test 2, the true positive fraction is

$$\text{tpf}2=\varphi_{.1}\theta_{.1}/(\varphi_{.1}\theta_{.1}+\varphi_{.0}\theta_{.0})$$

and the false positive fraction is

(10) $$\text{fpf}2=(1-\varphi_{.1})\theta_{.1}/(1-\varphi_{.1}\theta_{.1}+\varphi_{.0}\theta_{.0})$$

The main focus of this section is on measuring the accuracy of the combined test in the presence of verification bias of both tests using the BN (believe the negative) and BP (believe the positive) principles.

Assume the BP principle is in effect, then the true positive fraction for the combined test is

(11) $$\text{tpfbp}=P[Y_{1}=1orY_{2}=1|\text{D}=1]$$

while

(12) $$\text{fpfbp}=P[Y_{1}=1orY_{2}=1|\text{D}=0]$$

Now assume the BN assumption is in effect, then the true positive fraction is

(13) $$\text{tpfbn}=P[Y_{1}=1,Y_{2}=1|\text{D}=1]$$

while

(14) $$\text{fpfbn}=P[Y_{1}=1,Y_{2}=1|\text{D}=0]$$

The above four accuracy measures can be expressed as follows: for the BP assumption,

(15) $$\text{tpfbp}=(\varphi_{11}\theta_{11}+\varphi_{01}\theta_{01}+\varphi_{10}\theta_{10})/P[D=1]$$

and

(16) $$\text{fpfbp}=((1-\varphi_{11})\theta_{11}+(1-\varphi_{01})\theta_{01}+(1-\varphi_{10})\theta_{10})/P[D=0]$$

where

(17) $$P[D=1]=\varphi_{11}\theta_{11}+\varphi_{01}\theta_{01}+\varphi_{10}\theta_{10}+\varphi_{00}\theta_{00})$$

For the BN assumption

(18) $$\text{tpfbn}=\varphi_{11}\theta_{11}/P[D=1]$$

and

(19) $$\text{fpfbn}=(1-\varphi_{11})\theta_{11}/P[D=0]$$

Formulas (16)–(19) measure the combined accuracy of two binary tests assuming MAR and assuming an improper prior. If a uniform prior distribution is assumed for the *φ_ij_* and *θ_ij_*, adjust the hyper parameters in formulas (6) and (7) accordingly. For additional information about estimating accuracy with verification bias see Zhou [9,10] and Zhou and Castelluccio [11].

## 5. Example of MRI and CT to Assess Risk of Lung Cancer

Consider the hypothetical results of two correlated binary tests when verification bias is present as given below. The first test Y_1_ give the results for a CT determination of lung cancer risk, where a 0 indicates a small risk and a 1 a high risk of lung cancer, while the second test Y_2_ is a determination of lung cancer risk using MRI. The patients where D = 0 do not have lung cancer.

Using **BUGS CODE 1**, an analysis that determines the accuracy of the CT and MRI separately and for the combined accuracy is executed with 45,000 observations, with a burn in of 5,000 and a refresh of 100. The list statement of the code gives the data for this example, assuming an improper prior distribution:

### BUGS CODE 1

model; {
# two binary tests verification bias
# accuracy of combined tests
g00∼dgamma(m00,2)
g01∼dgamma(m01,2)
g10∼dgamma(m10,2)
g11∼dgamma(m11,2)
h<-g00+g01+g10+g11
th00<-g00/h
th01<-g01/h
th10<-g10/h
th11<-g11/h
ph00∼dbeta(s00,r00)
ph01∼dbeta(s01,r01)
ph10∼dbeta(s10,r10)
ph11∼dbeta(s11,r11)
s1.<-s11+s10
r1.<-r11+r10
s.1<-s01+s11
r.1<-r01+r11
r0.<-r00+r01
s0.<-s00+s01
s.0<-s00+s10
r.0<-r00+r10
ph1.∼dbeta(s1.,r1.)
ph.1∼dbeta(s.1,r.1)
ph0.∼dbeta(s0.,r0.)
ph.0∼dbeta(s.0,r.0)
th1.<-th11+th10
th.1<-th01+th11
th0.<-th01+th00
th.0<-th00+th10
# accuracy for test 1
tpf1<-ph1.*th1./pd1
fpf1<-(1-ph1.)*th1./(1-pd1)
# p[D-1]
pd1<-ph1.*th1.+ph0.*th0.
# accuracy for test 2
tpf2<-ph.1*th.1/pd2
fpf2<-(1-ph.1)*th.1/(1-pd2)
pd2<-ph.1*th.1+ph.0*th.0
# accuracy combined tests
# believe the positive, BP
tpfbp<-(ph11*th11+ph01*th01+ph10*th10)/pd
# P[d=1]
pd<-ph11*th11+ph10*th10+ph01*th01+ph00*th00
fpfbp<-((1-ph11)*th11+(1-ph10)*th10+(1-ph01)*th01)/(1-pd)
# believe the negative, BN
tpfbn<-ph11*th11/pd
fpfbn<- (1-ph11)*th11/(1-pd)}
# CT and MRI for lung cancer risk with improper prior
# for a uniform prior, add a one to the values in the list statement
list(s00=3,r00=18,s01=9,r01=13,s10=12,r10=9,s11=14,r11=4, m00=31,m01=31,m10=29,m11=25)
# activate initial values from the specification tool with the gen inits button

Note the above code closely follows the derivation given in formulas (1)–(19). This analysis can be executed by downloading the code from http://medtestacc.blogspot.com.

The results of the analysis show that the false positive fractions for the two modalities are fairly high being 0.286 and 0.38 for CT and MRI respectively, but on the other hand, the true positive fractions for the two modalities are somewhat low at 0.6755 and 0.60 respectively for CT and MRI. It is also observed that the MCMC errors for all parameters are less than 0.0001 and the posterior distributions of all parameters appear to be symmetric about the posterior mean. Assuming the BN rule, the true and false positive fractions are estimated as 0.3663 and 0.0880, but as for the BP rule, the corresponding estimates (based on the posterior mean) are 0.9165 and 0.5766! The fact that both modalities are not very accurate is reflected in the estimated combined accuracies for the BN and BP rules. At first glance, the BP rule is encouraging in that the true positive fraction is 0.9165, but the false positive fraction is also large as 0.5766. The BN rule gives a low estimate of 0.0880 for the false positive rate, but also a low estimate of 0.3663 for the true positive fraction. The posterior density of the true positive fraction for the BP rule is depicted below. Note what rule the user adopts depends on their personal preference.

## 6. Extreme Verification Bias

The reader is referred to Pepe ([7], p. 180) and Broemeling ([12], p. 166) for a description of many studies for those cases where when the test is positive, the subject is referred to the gold standard (verification of disease status), but if the test is negative the subject is not referred to the gold standard. In such situations, the true and false positive rates are not estimable. Consider the following hypothetical example given by above in Table 2, where MRI and CT are jointly used to detect lung cancer, where the results of CT are given by the first test and those of MRI given by *Y*_2_. Note when the subject is verified with D = 0, the patient does not have lung cancer.

The results of Table 2 have been modified so that all patients are referred to the gold standard (biopsy) except those where both tests are negative. There are 82 patients, of which 61 are verified for disease, namely the 18 who test positive when both CT and MRI are positive, 21 patients who test positive with CT but negative with MRI, and lastly 22 patients who test positive with MRI and negative with CT.

When both tests are negative, 21 patients are not subject to the gold standard. Since they were not verified, one does not know their disease status (D = 0 or D = 1), thus one does not know the fraction of patients with disease, and the true and false positive fractions cannot be estimated. This is a case where it is not possible to estimate the true and false positive fractions for either test. However, not all is lost because other measures of test accuracies for both tests can be estimated.

Consider the detection probability for CT namely P[*Y*_1_= 1, D = 1], then the estimated detection probability is 26/82 = 0.317, while the detection probability of MRI is estimated as 23/82 = 0.280. In lieu of the true and false positive fractions, the detection probability and the false referral probability for both tests aid in the estimation of the test accuracy of the combined tests. For example, the false referral probability for CT is P[*Y*_1_ = 1, D = 0] which is estimated as 13/82 = 0.158, and for MRI the false referral probability is estimated as 17/82 = 0.2073.

It is interesting to note that the detection probability of a test is expressed as

(20) DP=ρTPF

and the false referral probability as

(21) FRP=(1−ρ)FPF

where *ρ* = P[D = 1], is the probability of disease, and TPF and FPF are the true and false positive fractions respectively. From a given study the probability of disease cannot be estimated, however the true positive fractions and be compared with the ratio:

(22) $$\text{rtpf}(\text{ct}/\text{mri})=DP_{1}/DP_{2}$$

where *DP*_1_ is the detection probability of the first test and *DP*_2_ the detection probability for the second test. In a similar way, the false positive fractions can be compared by the ratio:

(23) $$\text{rtpf}(\text{ct}/\text{mri})=FRP_{1}/FRP_{2}$$

where *FRP*_1_ is the false referral probability for the first test and *FRP*_2_ the false referral probability of the second. Of course, the Bayesian approach will determine the posterior distribution of these quantities.

It is interesting to observe that the individual true and false positive fractions cannot be estimated, but that the true and false positive fractions of two tests can be compared for studies with extreme verification bias.

How is the accuracy of the combined tests estimated? Can one employ the BP (believe the positive) and BN rules to estimate the accuracy of the combined tests? The answer is no!

Consider the BP rule and refer to Table 4, where the true positive fraction of the combined tests is measured by P[*Y*_1_ =1*orY*_2_ =1|D = 1], however, the disease frequency cannot be estimated. On the other hand, the ratio of the true positive fraction for the BP rule relative to the true positive fraction of the BN rule can be measured by

(24) $$\begin{array}{c} \text{tpfbp}/\text{tpfbn}=\text{P}[Y_{1}=1orY_{2}=1|\text{D}=1]/\text{P}[Y_{1}=1\text{and}Y_{2}=1|\text{D}=1]\\ =P[Y_{1}=1orY_{2}=1\text{and D}=1]/P[Y_{1}=1\text{and}Y_{2}=1\text{and D}=1] \end{array}$$

From Table 4 for the CT-MRI study of lung cancer risk, a naïve estimate of this quantity is 35/14 = 2.5, which implies the true positive fraction for the BP rule is approximately 2.5 times larger than the true positive fraction for the BN rule.

In a similar way the false positive fraction for the BP rule relative to the false positive fraction of the BN rule can be measured as

(25) $$\begin{array}{c} \text{fpfbp}/\text{fpfbn}=\text{P}[Y_{1}=1orY_{2}=1|\text{D}=0]/\text{P}[Y_{1}=1\text{and}Y_{2}=1|\text{D}=0]\\ =P[Y_{1}=1orY_{2}=1\text{and D}=0]/P[Y_{1}=1\text{and}Y_{2}=1\text{and D}=0] \end{array}$$

Referring to Table 4 provides an estimate of 26/4 = 6.5 for comparing the false positive fractions for the two rules, implying the false positive rate for the BP rule is six and half times larger that of the BN rule.

Extreme verification bias does not provide sufficient information to estimate the usual measures of test accuracy, however, in lieu of those measures, it is possible to assess the accuracy of two binary tests (with extreme verification bias) by: (1) the detection probabilities; (2) the false referral probabilities and (3) the ratio of the true positive fraction for the BP rule relative to that of the BN rule, and (4) the ratio of the false positive fraction of the BP rule relative to that of the BN rule.

## 7. Bayesian Analysis for Extreme Verification Bias

The foundation of the Bayesian analysis given by formulas (1)–(19) which are now expanded to determine the posterior distribution of the detection probabilities and false referral probabilities of both tests.

With regard to estimating the combined accuracy of the two tests in the presence of extreme verification bias, the posterior distributions of the ratio of the true positive fraction of the BP relative to that of the BN rule is determined as is the ratio of the false positive fraction of the BP rule relative to that of the BN rule.

For the detection probability of the first test

(26) $$\text{DP}1=\text{P}[Y_{1}=1,D=1]=\theta_{1.}\varphi_{1.}$$

where the summation is taken over the missing subscript denoted by a period. Note the *θ_ij_* and *φ_ij_* are defined by formulas (1) and (2), thus in a similar way it can be shown that

(27) $$\text{DP}2=\text{P}[Y_{2}=1,D=1]=\theta_{.1}\varphi_{.1}$$

As for the false referral probabilities, the one for the first test is

(28) $$\text{FRP}1=P[Y_{1}=1,D=0]=(1-\varphi_{1.})\theta_{1.}$$

It can also be shown that for the second test,

(29) $$\text{FRP}2=\text{P}[Y_{2}=1,D=0]=(1-\varphi_{.1})\theta_{.1}$$

Referring to formulas (1), (2), (24), and (25), the ratio of the true positive fraction of the BP rule relative to that of the BN rule is

(30) $$\text{rtpfbp}/\text{tpfbn}=[\theta_{11}\varphi_{11}+\theta_{10}\varphi_{10}+\theta_{01}\varphi_{01}]/[\theta_{11}\varphi_{11}].$$

As for the ratio of the false positive fraction of the BP rule relative to that of the BN rule, it can be shown

(31) $$\text{rfpfbp}/\text{fpfbn}=[\theta_{11}(1-\varphi_{11})+\theta_{10}(1-\varphi_{10})+\theta_{01}(1-\varphi_{01})]/[\theta_{11}(1-\varphi_{11})].$$

Formulas (26)–(31) measure the accuracy of two combined binary tests when extreme verification bias is present.

## 8. Bayesian Analysis for Risk of Lung Cancer

**BUGS CODE 1** is amended with the following WinBUGS® code.

For the detection probabilities, the statements are:

(32) DP1<−th1.∗ph1.

and

(33) DP2<−th.1∗ph.1

For the false referral probabilities of the two tests:

(34) FRP1<−(1−ph1.)∗th1.

and

(35) FRP2<−(1−ph.1)∗th.1.

The above code corresponds to formulas (26)–(20).

Refer to formula (30) for the ratio of the true positive fraction of the BP rule relative to that of the BN rule, thus the following:

Rtpfbptpfbn<−(th11∗ph11+th10∗ph10+th01∗ph01)/(th11∗ph11)

Refer to formula (31) for the ratio of the false positive fraction of the BP rule to that of the BN rule, thus the code is

Rfpfbpfpfbn<−(th11∗(1−ph11)+th10∗(1−ph10)+th01∗(1−ph01))/(th11∗(1−ph11))

The list statement for the data is given by:

list(s00=1,r00=1,s01=9,r01=13,s10=12,r10=9,s11=14,r11=4,m00=21,m01=22,m10=21,m11=18)

which assumes an improper prior density for all parameters. Although s00 = 1 and r00 = 1 are zero (when the two tests are negative all patients are referred to the gold standard), I put a one, which does not affect the analysis.

The analysis for the CT-MRI determination of lung cancer risk with extreme verification bias is based on the amended **BUGS CODE 1** by generating 55,000 samples from the posterior distributions of the detection and false referral probabilities of both CT and MRI. Also computed are the posterior distributions of the ratio of the true and false positive fractions of the BP rule relative to that of the BN rule. I used a burn in of 5,000 observations with a refresh of 100 to give.

**Figure 2:**
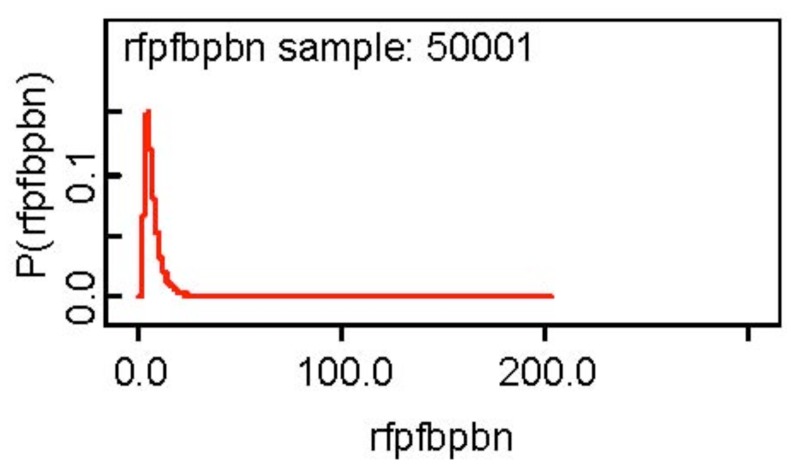
Posterior density of the ratio of the false positive fraction. BP relative to BN.

One notices the skewness of the posterior distribution of the ratio of the false positive fraction of the BP rule relative to the BN rule and the skewness is evident from Figure 2 below. I would use 6.902 the posterior median as a point estimate of the ratio.

The other posterior distributions appear to be symmetric about the posterior mean. The detection probability of the two tests are similar, but the false referral probability of CT is somewhat less than that of MRI and the TPF of the BP rule appears to be 2.61 times that of the BN rules. On the other hand, the false positive fraction of the BP rule is 6.9 times that of the BN rule.

Recall that the true and false positive fractions of the two rules are not known, thus, it is difficult to interpret these ratios. The detection probability of a test is somewhat related to the true positive fraction in that one would favor a test with a higher detection probability. Also, of course, one would favor a test with lower false referral probability, thus, the overall conclusion about test comparison is to favor the CT determination of lung cancer risk. As for the accuracy of the combined test, the two ratios one for the true positive fraction and one for the false positive fraction provide the relevant information. Should the accuracy be based on the BP or the BN rule? The BP rule gives a larger true positive fraction, but unfortunately a much larger false positive fraction.

## 9. Verification Bias for Two Ordinal Tests

When analyzing the accuracy of combined tests when the tests are ordinal, a somewhat different approach is taken, where the overall accuracy is measured by the area of the ROC curve of the risk score. The risk scores are the probability of disease of each patient, usually determined. An informative presentation of the risk score is given by Pepe ([7], p. 271).

Our general methodology is based on inverse probability weighting which transforms the original table of observations with verification (See Table), to an imputed table. Such ideas will be explained in a later section, but the reader is referred to Pepe ([7], p.172) and Broemeling ([13], p. 279) for additional details of the inverse probability weighting approach for estimating the accuracy of tests with verification bias.

The analysis of assessing the accuracy of two tests is now expanded to include two ordinal tests *T*_1_ and *T*_2_, where the general layout is given by Table 5. As before the *s_i_* denote the number of patients for the 9 events when D = 1, while the *r_i_* represent the number of cases for the nine events when D = 0. The total number of observations is $\overset{i=9}{\underset{i=1}{\text{∑}}}m_{i}$. Each test has three values, but of course the general situation has a similar scenario.

In what is to follow, the posterior distribution of the ROC area of the two ordinal tests is developed, which is to be followed by an explanation of inverse probability weighting, and lastly the use of the risk score to asses the accuracy of the combined tests is explained.

## 10. Posterior Distribution of the ROC Areas for Two Ordinal Tests with Verification Bias

Recall from formulas (1) and (2) that

$$\varphi_{ij}=P[D=1|Y_{1}=i,Y_{2}=j]\;\text{and}\;\theta_{ij}=P[Y_{1}=i,Y_{2}=j]$$

thus

(36) $$\varphi_{i}.=P[D=1|Y_{1}=i]$$

and

(37) $$\varphi_{.i}=P[D=1|Y_{2}=i]$$

for i = 1,2,3, then assuming an improper prior density,

(38) $$\varphi_{i.}\sim \text{beta}(s_{i.},r_{i.})$$

and

(39) $$\varphi_{.i}\sim \text{beta}(s_{.i},r_{.i})$$

The improper prior density used here is the reciprocal of the parameters over the relevant region of the parameter space.

If uniform prior is appropriate, add a 1 to the hyper parameters of the *φ_i_* and *θ_i_* for i = 1,2,3.

Note that

$$\begin{array}{c} s_{1.}=s_{1}+s_{2}+s_{3}\\ s_{.1}=s_{1}+s_{4}+s_{7}\\ r_{1.}=r_{1}+r_{2}+r_{3}\;\text{etc}. \end{array}$$

Also, let

(40) $$\theta_{1}=P[Y_{1}=1,Y_{2}=1]$$

and

(41) $$\theta_{9}=P[Y_{1}=3,Y_{2}=3]$$

In addition, let

$$\theta_{1.}=\theta_{1}+\theta_{2}+\theta_{3}$$

thus

(42) $$\theta_{1.}=P[Y_{1}=1]$$

Also let

$$\theta_{.3}=\theta_{3}+\theta_{6}+\theta_{9}$$

then

(43) $$\theta_{.3}=P[Y_{2}=3]\text{etc}.$$

In order to compute the area under the ROC curve, Bayes theorem is used to compute

$$\alpha 1_{i}=\varphi_{i.}\theta_{i.}/t\alpha 1$$

where i = 1,2,3, and

$$t\alpha 1=\sum_{i=1}^{i=3}\varphi_{i.}\theta_{i.}$$

Note that

(44) $$\alpha 1_{i}=P[Y_{1}=i|D=1]$$

And in a similar manner

$$\beta 1_{i}=(1-\varphi_{i.})\theta_{i.}/t\beta 1$$

where

$$t\beta 1=\sum_{i=1}^{i=3}(1-\varphi_{i.})\theta_{i.}$$

thus

(45) $$\beta 1_{i}=P[Y_{1}=i|\text{D}=0]$$

(46) $$\alpha 2_{i}=P[Y_{2}=i|\text{D}=1]$$

and

(47) $$\beta 2_{i}=P[Y_{2}=i|\text{D}=0]$$

can be defined. The ROC area for the first test is

(48) $$A_{1}=A_{11}+A_{12}$$

where

(49) $$A_{11}=\alpha 1_{2}\beta 1_{1}+\alpha 1_{3}(\beta 1_{1}+\beta 1_{2})$$

and

(50) $$2A_{12}=\sum_{i=1}^{i=3}\alpha 1_{i}\beta 1_{i}$$

Of course, a similar expression holds for the ROC area of test 2. After determining the posterior distribution of the ROC areas of the two tests, the accuracy of the combined tests will be measured by the ROC area of the risk score. In order to illustrate Bayesian inference for estimating the accuracy of two ordinal tests, consider the following example, two readers are diagnosing breast cancer. They are both employed by a university cancer center and they both use a three–point scale to diagnose the disease, where a 1 indicates definitely no lesion is seen in the mammogram, a 2 denotes there is a possibility that a lesion is present, and a 3 signifies that a lesion is definitely present The diseased patients in fact have breast cancer and the non-diseased definitely do not have breast cancer.

The total number of patients is $\overset{i=9}{\underset{i=1}{\text{∑}}}m_{i}=1,768$, and for each of the nine categories patients are not referred to the gold standard, for example. For example, when *T*_1_ = *T*_2_ = 1, 109 are referred to the gold standard and 2 are not, while if *T*_1_ = *T*_2_ = 2, 83 are not referred, while 153 are subject the gold standard. What are the ROC areas of the two tests? The following code is based on the previous development of the posterior distributions of the relevant parameters (given by formulas (36)–(50)) and appears below.

### BUGS CODE 2

model;
# hypothetical data set
# two tests for staging melanoma
# one rater is a surgeon the other a dermatologist
# ratings are: stage 1, stage 2, stage 3
# similar to Zhou([8],p347) on CT and MRI
{
for (i in 1:9) {ph[i]∼dbeta(s[i],r[i])}
for (i in 1:9) {g[i]∼dgamma(m[i],2)}
ms<-sum(g[])
for (i in 1:9) {theta[i]<-g[i]/ms }
theta1.<- theta[1]+theta[2]+theta[3]
theta2.<- theta[4]+theta[5]+theta[6]
theta3.<- theta[7]+theta[8]+theta[9]
theta.1<- theta[1]+theta[4]+theta[7]
theta.2<- theta[2]+theta[5]+theta[8]
theta.3<- theta[3]+theta[6]+theta[9]
s1.<-s[1]+s[2]+s[3]
s2.<-s[4]+s[5]+s[6]
s3.<-s[7]+s[8]+s[9]
s.1<-s[1]+s[4]+s[7]
s.2<-s[2]+s[5]+s[8]
s.3<-s[3]+s[6]+s[9]
r1.<-r[1]+r[2]+r[3]
r2.<-r[4]+r[5]+r[6]
r3.<-r[7]+r[8]+r[9]
r.1<-r[1]+r[4]+r[7]
r.2<-r[2]+r[5]+r[8]
r.3<-r[3]+r[6]+r[9]
# the prob D=1 given Y1=1
ph1.∼dbeta(s1.,r1.)
ph2.∼dbeta(s2.,r2.)
ph3.∼dbeta(s3.,r3.)
ph.1∼dbeta(s.1,r.1)
ph.2∼dbeta(s.2,r.2)
ph.3∼dbeta(s.3,r.3)
# the prob the first test =1 given d=1
alpha1[1]<- ph1.*theta1./dalpha1
alpha1[2]<- ph2.*theta2./dalpha1
alpha1[3]<- ph3.*theta3./dalpha1
dalpha1<- ph1.*theta1.+ph2.*theta2.+ph3.*theta3.
# the prob the first test =1 given D=0
beta1[1]<-((1-ph1.)*theta1.)/dbeta1
beta1[2]<-((1-ph2.)*theta2.)/dbeta1
beta1[3]<-((1-ph3.)*theta3.)/dbeta1
dbeta1<-(1-ph1.)*theta1.+(1-ph2.)*theta2.+(1-ph3.)*theta3.
# the prob that the second test =1 given d=1
alpha2[1]<- ph.1*theta.1/dalpha2
alpha2[2]<- ph.2*theta.2/dalpha2
alpha2[3]<- ph.3*theta.3/dalpha2

dalpha2<- ph.1*theta.1+ph.2*theta.2+ph.3*theta.3
beta2[1]<-((1-ph.1)*theta.1)/dbeta2
beta2[2]<-((1-ph.2)*theta.2)/dbeta2
beta2[3]<-((1-ph.3)*theta.3)/dbeta2
dbeta2<-(1-ph.1)*theta.1+(1-ph.2)*theta.2+(1-ph.3)*theta.3
# area of test 1
A1<- A11+A12
A11<- alpha1[2]*beta1[1]+alpha1[3]*(beta1[1]+beta1[2])
A12<- (alpha1[1]*beta1[1]+alpha1[2]*beta1[2]+alpha1[3]*beta1[3])/2
# area of test 2
A2<- A21+A22
A21<- alpha2[2]*beta2[1]+alpha2[3]*(beta2[1]+beta2[2])
A22<- (alpha2[1]*beta2[1]+alpha2[2]*beta2[2]+alpha2[3]*beta2[3])/2
d<-A1-A2
}
# two readers
# assume an improper prior
# see Table 6
list(r = c(101,105,83,67,72,40,41,30,4), s = c(8,26,51,43,81,94,117,140,208), m= c(111,149,196,124,236,201,221,210,320))
# the initial values are activated from the specification tool by clicking on the gen inits button.

The code closely follows formulas (36)–(50), and the analysis is executed with 55,000 samples from the posterior distribution of the parameters, with a burn in of 5,000 and a refresh of 100 and the results are reported in Table 7.

Reader 1 appears more accurate that reader 2. It should be noted that the gold standard is biopsy of the tissue from the suspected lesion. The MCMC errors are quite “small” and give one confidence that the simulation is providing accurate estimates of the accuracy.

The main goal of the analysis is to estimate the combined accuracy of the two readers nd compare it the individual estimated ROC areas given by Table 7.

## 11. Inverse Probability Weighting

Pepe ([7], p. 171) describes an interesting variation on estimating test accuracy with verification bias by the inverse probability weighting technique, and for further information, see Begg and Greenes [14]. Briefly this method involves constructing an imputed data table from the observed data table with verification bias. Consider the observed data table (Table 6) with verification bias. For each pair of values (*T*_1_,*T*_2_), the cell entries for *s_i_* and *r_i_* are multiplied by the inverse of the verification rate. For example, when (*T*_1_,*T*_2_) = (1,1) the verification rate is 109/111, thus for *s*_1_, 8 is replaced 8(111/109) = 8.146 and for *r*_1_, the number 101 is replaced by 101(111/109) = 102.85. If this is done for the remaining 8 events, the observed Table 6 is replaced by the selected table below:

The imputed table has no verification bias and the ROC areas of the two tests based on this table are the same as those based on Table 6, the table with verification bias. In general it can be shown for binary tests that the true and false positive fractions are the same, for those based on the observed table and those based on the selected table without verification bias. See Pepe ([7], p. 172), thus, inverse probability weighting is appropriate for two ordinal tests with verification bias. It is seen the effect of inverse probability weighting is to convert a table with verification bias into a table without. The presentation is continued by developing the risk score to assess the accuracy of two ordinal tests with verification bias, where the basic idea is to compute the ROC of the risk score. For additional information about estimating the test accuracy of ordinal tests with verification bias using the ROC area, see Gray *et al.* [15].

## 12. The Risk Score

The risk score is the probability that D = 1, which is the value assigned to all patients in the study. For example, for each of the 1,768 patients in the melanoma staging study portrayed in Table 8, the probability of disease is assigned and the ROC area computed. The ROC area of the risk score is the combined accuracy of the two ordinal tests with verification bias.

The risk score is defined as

(51) RS(Y)=P[D=1∣Y]

and has the property that it is a monotone function of the likelihood ratio. Simply stated, the risk score assigns a probability of disease to each study subject.

The risk score (51) has the same ROC curve as the likelihood ratio and the same optimal properties. Observe that

(52) $$\begin{array}{c} \text{RS}(\text{Y})=\text{P}[\text{D}=1|\text{Y}]\\ =\text{P}[\text{Y}|\text{D}=1]\text{P}[\text{D}=1]/\text{P}[\text{Y}]\\ =\text{P}[\text{Y}|\text{D}=1]\text{P}[\text{D}=1]/\{\text{P}[\text{Y}|\text{D}=1]\text{P}[\text{D}=1]+\text{P}[\text{Y}|\text{D}=0]\text{P}[\text{D}=0]\}\\ =\text{LR}(\text{Y})\text{P}[\text{D}=1]/\{\text{LR}(\text{Y})\text{P}[\text{D}=1]+\text{P}[\text{D}=1]\} \end{array}$$

which shows that the risk score is a monotone increasing function of the likelihood ratio, which implies that the ROC curve of risk score is the same as that of the likelihood ratio. For our purposes the risk score will be used to measure the accuracy of combined tests, namely, using the area of the ROC curve of the risk score. Pepe ([7], p. 274) shows the utility of logistic regression for finding the ROC curve of the risk score. Note that the following statements show why.

Suppose the risk score is expressed as

(53) logitP[D=1∣Y]=γ+g(λ,Y)

where g is a known function, then:

- the parameter *λ* can be estimated, even for retrospective designs in which the sampling depends on D, and
- the function g is optimal for determining the ROC curve of the risk function.

From a practical point of view, logistic regression can be used to determine the ROC curve of the risk function, but it should be noted that finding a suitable function g can be a challenge. After all, g can be a complicated non-linear function of λ and/ or Y, but it would be convenient if g is linear in the test scores Y. In order to estimate the logistic regression function or risk score, Broemeling ([13], p. 474), takes a Bayesian approach.

For the example of Table 8, where two readers are diagnosing breast cancer, the risk score is computed by logistic regression using the following code.

### BUGS CODE 3

model;
# logistic regression
{
for(i in 1:N){d[i]∼dbern(theta[i])}
for(i in 1:N){logit(theta[i])<-b[1]+b[2]*T1[i]+b[3]*T2[i]}
# prior distributions
for(i in 1:3){b[i]∼dnorm(0.000,.0001)}
}

In order to accommodate the data, a list statement must be added, which consists of three vectors, each of dimension 1,768, corresponding to the number of patients in the study: The vectors *T*_1_ and *T*_2_ will consist of the values 1,2, or 3 corresponding to Table 8, while a d vector will consists of zeros and ones, where a 1 indicates the patient has melanoma and a 0 indicates no melanoma, thus, the first 1,078 components of d consist of a one, while the remaining 690 consist of zeros. After execution of the **BUGS CODE 3**, with 45,000 samples from the posterior distribution, with a burn in of 5,000, and a refresh of 100, the posterior distribution of coefficients of the logistic regression is given in Table 9.

The posterior distributions indicate the coefficients are not zero and are important in determining the risk score of each patient, but the main interest is in the vector theta (of dimension 1,768) where the median of each component is used as the risk score. There were 9 distinct risk scores: 0.086, 0.187, 0.36, 0.336, 0.5542, 0.733, 0.7542, 0.8704, and 0.9426. Therefore, I computed the ROC using the basic formula from Broemeling ([12], p. 58) for the ROC area of a test with ordinal responses.

**Using BUGS CODE 4**, I computed the ROC area of the risk score via **BUGS CODE 4** with posterior mean 0.842 with a 95% credible interval of (0.8238, 0.8608). (Also, the ROC area was computed with a non-parametric technique with SPSS with the same result as the Bayesian). Thus, using the risk score, the estimated ROC area is 0.842 compared to a ROC area of 0.78 for the surgeon and 0.63 for the dermatologist, thus, the accuracy of the combining results of two readers is more than the individual accuracies. Consider the posterior analysis:

Based on **BUGS CODE 4**, the analysis is executed with 55,000 observations with a burn in of 5,000 and a refresh of 100. A2 is the probability of a tie, and auc is the ROC area. Small MCMC errors for the parameters are evident, and the posterior distribution of all parameters appear to be symmetric. The code below is explained with explanatory remarks indicated by #.

### BUGS CODE 4

# Area under the curve
# Ordinal values
# Nine values
model;
{
# generate Dirichlet distribution
g11∼dgamma(a11,2)
g12∼dgamma(a12,2)
g13∼dgamma(a13,2)
g14∼dgamma(a14,2)
g15∼dgamma(a15,2)
g16∼dgamma(a16,2)
g17∼dgamma(a17,2)
g18∼dgamma(a18,2)
g19∼dgamma(a19,2)
g01∼dgamma(a01,2)
g02∼dgamma(a02,2)
g03∼dgamma(a03,2)
g04∼dgamma(a04,2)
g05∼dgamma(a05,2)
g06∼dgamma(a06,2)
g07∼dgamma(a07,2)
g08∼dgamma(a08,2)
g09∼dgamma(a09,2)
g1<-g11+g12+g13+g14+g15+g16+g17+g18+g19
g0<-g01+g02+g03+g04+g05+g06+g07+g08+g09
# posterior distribution of probabilities for response of diseased patients
theta1<-g11/g1
theta2<-g12/g1
theta3<-g13/g1
theta4<-g14/g1
theta5<-g15/g1
theta6<-g16/g1
theta7<-g17/g1
theta8<-g18/g1
theta9<-g19/g1
# posterior distribution for probabilities of response of non-diseased patients
ph1<-g01/g0
ph2<-g02/g0
ph3<-g03/g0
ph4<-g04/g0
ph5<-g05/g0
ph6<-g06/g0
ph7<-g07/g0
ph8<-g08/g0
ph9<-g09/g0
# auc is area under ROC curve
#A1 is the P[Y>X]
#A2 is the P[Y=X]
auc<- A1+A2/2
A1<-theta2*ph1+theta3*(ph1+ph2)+theta4*(ph1+ph2+ph3)+
theta5*(ph1+ph2+ph3+ph4)+theta6*(ph1+ph2+ph3+ph4+ph5)+
theta7*(ph1+ph2+ph3+ph4+ph5+ph6)+
theta8*(ph1+ph2+ph3+ph4+ph5+ph6+ph7)+
theta9*(ph1+ph2+ph3+ph4+ph5+ph6+ph7+ph8)
A2<- theta1*ph1+theta2*ph2+theta3*ph3+theta4*ph4
+theta5*ph5+theta6*ph6+theta7*ph7+theta8*ph8+theta9*ph9
}
#see Table 8
# diagnosing breast cancer with two readers
# verification bias
# improper prior
list(a11=8,a12=30,a13=75,a14=48,a15=.125,a16=164,a17=141,a18=173,a19=314,a01=103,a02=119,a 03=121,a04=76,a05=111,a06=57,a07=60,a08=37,a09=6)

# initial values
list(g11=1,g12=1,g13=1,g14=1,g15=1,g16=1,g17=1,g18=1,g19=1,g01=1,g02=1,g03=1, g04=1,g05=1, g06=1,g07=1,g08=1,g09=1)

## 13. Comments and Conclusions

This presentation has reviewed the Bayesian methodology available for estimating the test accuracy when two or more tests are used to diagnose disease. The main focus is on tests that are subject to verification bias, that is, when some of the patients are not subject to the gold standard (are not verified for disease status). When verification bias occurs certain techniques are employed to correct for bias. If the usual estimators are calculated only for those patients that are verified for disease, the estimators are biased, thus, this article develops Bayesian procedures that “correct” for bias. By imposing the missing at random assumption, Bayesian estimators of the usual measures of test accuracy are developed. For two binary tests, the true and false positive fractions estimate the test accuracy, while for two ordinal tests the ROC area of the score function estimates the test accuracy of the combined tests. An interesting variation of verification bias is extreme verification bias, which is present when all of the patients that test negative with both tests are not verified for disease. In such a scenario, the true and false positive fractions cannot be estimated. However, other measures of the combined accuracy are available and easily estimated with Bayesian inference. Bayesian inference is illustrated with examples involving the diagnosis of breast and lung cancer.

There is a large literature on the subject of verification bias, and for additional recent information about the subject see [16-19]. Very little has appeared from a Bayesian viewpoint, however, Buzoianu and Kadane [20] present results on adjustment for verification bias. Broemeling [12,13] is the only book that focuses on the Bayesian approach to verification bias.

## Figures and Tables

**Figure 1:**
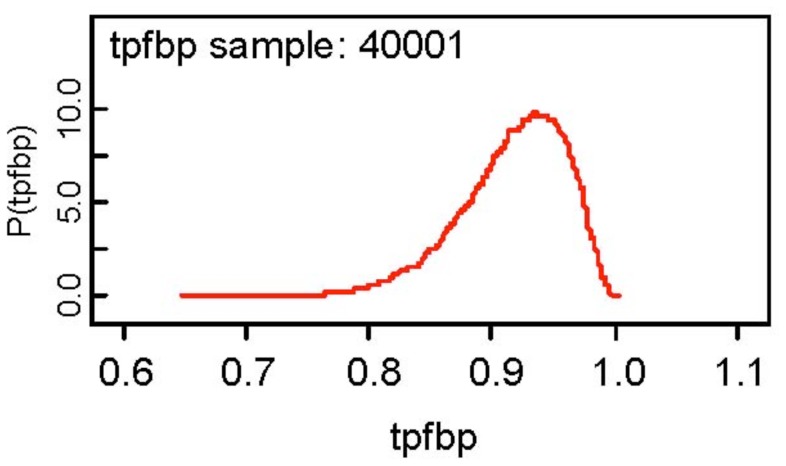
Posterior density of true positive fraction BP rule.

**Table 1:** Two binary scores with verification bias.

Y_1_ = 1, 0				
V = 1	Y_2_ = 1	Y_2_ = 0	Y_2_ = 1	Y_2_ = 0
D = 1	*s*_11_	*s*_10_	*s*_01_	*s*_00_
D = 0	*r*_11_	*r*_10_	*r*_01_	*r*_00_
V = 0	*u*_11_	*u*_10_	*u*_01_	*u*_00_
Total	*m*_11_	*m*_10_	*m*_01_	*m*_00_

**Table 2:** CT and lung cancer risk.

Y_1_ = 1, 0				
V = 1	Y_2_ = 1	Y_2_ = 0	Y_2_ = 1	Y_2_ = 0
D = 1	*s*_11_ = 14	*s*_10_ = 12	*s*_01_ = 9	*s*_00_ = 3
D = 0	*r*_11_ = 4	*r*_10_ = 9	*r*_01_ = 13	*r*_00_ = 18
V = 0	*u*_11_ = 7	*u*_10_ = 8	*u*_01_ = 9	*u*_00_ = 10
Total	*m*_11_ = 25	*m*_10_ = 29	*m*_01_ = 31	*m*_00_ = 31

**Table 3:** Posterior analysis for the CT and MRI determination of lung cancer risk with verification bias.

**Parameter**	**Mean**	**SD**	**Error**	**2 1/2**	**Median**	**97 1/2**
fpf1	0.2864	0.06201	<0.0001	0.1725	0.284	0.4141
fpf2	0.3809	0.0668	<0.0001	0.253	0.3799	0.5144
fpfbn	0.0880	0.0697	<0.0001	0.0264	0.0829	0.1795
fpfbp	0.5766	0.0653	<0.0001	0.4559	0.5777	0.7021
tpf1	0.6755	0.0712	<0.0001	0.5305	0.6779	0.8074
tpf2	0.6007	0.0737	<0.0001	0.4538	0.6018	0.7414
tpfbn	0.3663	0.0694	<0.0001	0.2367	0.3645	0.5081
tpfbp	0.9165	0.0439	<0.0001	0.8131	0.9233	0.9812

**Table 4:** CT and MRI for lung cancer risk with extreme verification bias.

Y_1_ = 1, 0				
V = 1	Y_2_ = 1	Y_2_ = 0	Y_2_ = 1	Y_2_ = 0
D = 1	*s*_11_ = 14	*s*_10_ = 12	*s*_01_ = 9	*s*_00_ = 0
D = 0	*r*_11_ = 4	*r*_10_ = 9	*r*_01_ = 13	*r*_00_ = 0
V = 0	*u*_11_ = 0	*u*_10_ = 0	*u*_01_ = 0	*u*_00_ = 21
Total	*m*_11_ = 18	*m*_10_ = 21	*m*_01_ = 22	*m*_00_ = 21

**Table 5:** Bayesian analysis for extreme verification bias (callout).

**Parameter**	**Mean**	**SD**	**Error**	**2 1/2**	**Median**	**97 1/2**
DP1	0.3169	0.0508	<0.0001	0.22	0.3155	0.4206
DP2	0.2805	0.0493	<0.0001	0.1891	0.2787	0.3823
FRP1	0.1586	0.0400	<0.0001	0.0882	0.1559	0.2411
FRP2	0.2074	0.0446	<0.0001	0.127	0.2051	0.3016
Ratio TPF	2.618	0.5922	0.0019	1.774	2.515	4.503
Ratio FPF	8.346	5.629	0.0168	3.187	6.902	22.01

**Table 6:** Diagnosing breast cancer with two readers.

*T*_1_ = 1, 2, 3									
*T*_2_	1	2	3	1	2	3	1	2	3
D = 1	*s*_1_ 8	*s*_2_ 26	*s*_3_ 51	*s*_4_ 43	*s*_5_ 81	*s*_6_ 94	*s*_7_ 117	*s*_8_ 140	*s*_9_ 208
D = 0	*r*_1_ 101	*r*_2_ 105	*r*_3_ 83	*r*_4_ 67	*r*_5_ 72	*r*_6_ 40	*r*_7_ 41	*r*_8_ 30	*r*_9_ 4
V = 0	*u*_1_ 2	*u*_2_ 18	*u*_3_ 62	*u*_4_ 14	*u*_5_ 83	*u*_6_ 67	*u*_7_ 63	*u*_8_ 40	*u*_9_ 108
Total	*m*_1_ 111	*m*_2_ 149	*m*_3_ 196	*m*_4_ 224	*m*_5_ 236	*m*_6_ 201	*m*_7_ 221	*m*_8_ 210	*m*_9_ 320

**Table 7:** Bayesian analysis for ROC areas of two readers.

Parameter	Mean	SD	Error	2 1/2	Median	97 1/2
A1(reader 1)	0.7867	0.0119	<0.00001	0.763	0.787	0.8097
A2(reader 2)	0.6351	0.0145	<0.00001	0.6062	0.6352	0.6633

**Table 8:** Diagnosing breast cancer with two readers imputed table via inverse probability weighting.

*T*_1_ = 1, 2, 3								
1	2	3	1	2	3	1	2	3
*s*_1_ 8	*s*_2_ 30	*s*_3_ 75	*s*_4_ 48	*s*_5_ 125	*s*_6_ 141	*s*_7_ 164	*s*_8_ 164	*s*_9_ 314
*r*_1_ 103	*r*_2_ 119	*r*_3_ 121	*r*_4_ 76	*r*_5_ 111	*r*_6_ 60	*r*_7_ 57	*r*_8_ 37	*r*_9_ 6
*u*_1_ 0	*u*_2_ 0	*u*_3_ 0	*u*_4_ 0	*u*_5_ 0	*u*_6_ 0	*u*_7_ 0	*u*_8_ 0	*u*_9_ 0
*m*_1_ 111	*m*_2_ 149	*m*_3_ 196	*m*_4_ 124	*m*_5_ 236	*m*_6_ 201	*m*_7_ 221	*m*_8_ 210	*m*_9_ 320

**Table 9:** Posterior distribution of the logistic regression the risk score.

**Parameter**	**Mean**	**SD**	**Error**	**2 1/2**	**Median**	**97 1/2**
b[1]	−4.947	0.2859	0.00173	−5,515	−4.943	−4.395
b[2]	1.688	0.0851	<0.0001	1.524	1.687	1.857
b[3]	0.8946	0.0804	<0.0001	0.7373	0.894	1.053

**Table 10:** Posterior analysis of combined accuracy breast cancer example with two readers.

**Parameter**	**Mean**	**SD**	**Error**	**2 1/2**	**Median**	**97 1/2**
A1	0.8099	0.0106	<0.00001	0.7885	0.8101	0.8308
A2	0.0657	0.0030	<0.00001	0.0599	0.0656	0.0716
auc	0.8428	0.0094	<0.00001	0.8238	0.843	0.8608
